# The occurrence rate and clinical application of Osteo-line on the femur neck

**DOI:** 10.1186/s13018-021-02289-6

**Published:** 2021-02-25

**Authors:** Mingchen Zou, Haotian Wu, Shuangquan Yao, Dong Ren, Song Liu, Yueju Liu, Zhaohui Song

**Affiliations:** 1 Department of Orthopedic Surgery, The 3rd Hospital of Hebei Medical University, Shijiazhuang, People’s Republic of China; 2 Key Laboratory of Biomechanics of Hebei Province, Shijiazhuang, People’s Republic of China

**Keywords:** Total hip arthroplasty, Osteo-line on femur neck, Leg length discrepancy, Osteotomy

## Abstract

**Background:**

This study was done to observe the incidence of Osteo-line on the femur neck and to explore the clinical application of Osteo-line in osteotomy.

**Methods:**

Eighty-nine adult femur specimens were selected to observe the incidence of Osteo-line on the femur neck. From August 2015 to January 2019, a total of 278 patients who completed unilateral hip arthroplasty at the Third Hospital of Hebei Medical University were retrospectively included. Patients who accepted osteotomy via Osteo-line on the femur neck were defined as the experimental group (*n* = 139), and patients who accepted osteotomy via traditional method (The femoral distance 1.5 cm above the trochanter was retained for osteotomy by visual inspection.) were defined as the control group (*n* = 139). According to the postoperative pelvic X-ray, Photoshop was used to evaluate the leg length discrepancy (LLD) by the CFR-T-LT method.

**Results:**

Among the 89 specimens, the incidence of anterior Osteo-line was 75.28%, and the incidence of posterior Osteo-line was 100%. According to the clinical application results, the incidence of anterior Osteo-line on the femur neck was 80%, and the incidence of posterior Osteo-line was 100%. The Osteo-line was clearer than those on the femoral specimens. Twenty-six cases had LLD greater than 1 cm (9.29%), including 2 cases in the experimental group and 24 cases in the control group. The average postoperative LLD in the experimental group (0.19 ± 0.38 mm) was significantly shorter than in the control group (0.54 ± 0.51 mm)(*P* = 0.005).

**Conclusion:**

The incidence of Osteo-line on the femur neck was high, and patients who accepted osteotomy via Osteo-line on the femur neck can achieve shorter postoperative LLD than the control group.

## Background

Total hip arthroplasty (THA) reduces pain and improves function in patients with end-stage arthritis of the hip and is associated with a high satisfaction rate and a low incidence of complications [1–3]. The plane between 1.25 ± 0.25 cm above the tip of the lesser trochanter and the line to the base of the femoral neck on the greater trochanter side are usually used as the femoral neck osteotomy position in THA [4]. However, this plane is subjectively and is limited by the surgical experience of different surgeons. Two complications after THA are hip instability and leg length discrepancy (LLD), among which significant LLD after THA is a cause for patient dissatisfaction and possible litigation [5, 6]. LLD induces functional complications (limping, low back pain, instability, and neurological sequelae) and significantly affects clinical score values when the length difference exceeds 10 mm [7, 8]. Thus, LLD is a common and serious complication that deserves careful attention.

Many scholars have adopted different surgical techniques in order to formulate more precise osteotomy plans and reduce the risk of unequal length of the lower limbs after hip replacement [9–11]. Hofmann et al. [12] reported leg length discrepancies (> 6 mm) can be minimized with an intraoperative X-ray. In each case, preoperative templating was carefully performed, an intraoperative pelvis X-ray was obtained to assess accuracy, and appropriate adjustments were made. However, it is still necessary to repeat the measurement during the operation to ensure that it matches the preoperative template, which increases the operation time and blood loss. Shiramizu et al. [13] designed a caliper to estimate limb lengthening during THA and enabled accurate measurement. However, the integrity of the femoral neck must be ensured, and it is not suitable for femoral neck fractures and other femoral neck diseases in which the length of the femoral neck has changed.

**Fig. 1:**
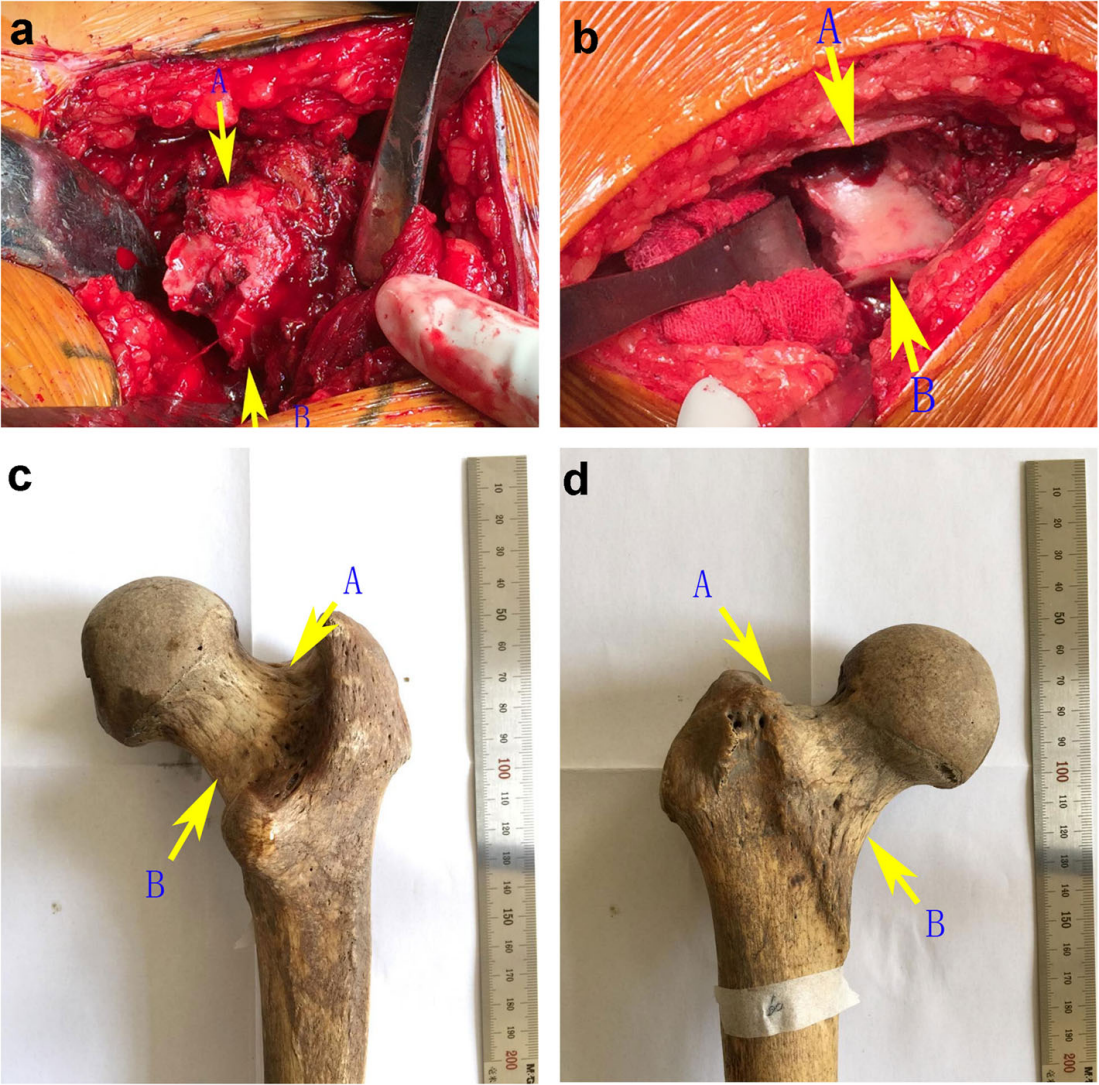
Osteo-line on femor neck. The top of Osteo-line (point A) is located exactly at the base of the femoral neck on the greater trochanter side, and the bottom of Osteo-line (point B) is located on the side of the lesser trochanter of the femur. The Osteo-line is within 1.25 ± 0.25 cm above the tip of the lesser trochanter

We found that during the transition of the cortical bone from the trochanteric part to the cortical bone of the femoral neck, an Osteo-line is naturally formed at the front and back of the base of the femoral neck (Fig. 1). The top of Osteo-line (point A) is located exactly at the base of the femoral neck on the greater trochanter side, the bottom of Osteo-line (point B) is located on the side of the lesser trochanter of the femur, and the Osteo-line is within 1.25 ± 0.25 cm above the tip of the lesser trochanter. Using the Osteo-line as an anatomical landmark for femoral neck osteotomy can effectively simplify the surgical steps of THA. The purpose of this study is to observe the occurrence rate of the Osteo-line and whether it can reduce the rates of LLD after THA in clinical application.

## Methods

### Femur specimens

Eighty-nine adult femur specimens from of Hebei Medical University were selected, of which 44 were left femur specimens and 45 were right femur specimens. The inclusion criteria were (1) dry adult femoral specimens with regular shape, complete shape, and good texture; (2) femoral specimens without cartilage. The exclusion criteria were specimens with previous proximal femoral fractures, previous bone metabolic diseases, previous deformities, and previous internal fixation.

### Osteo-line on femur neck

**Fig. 2:**
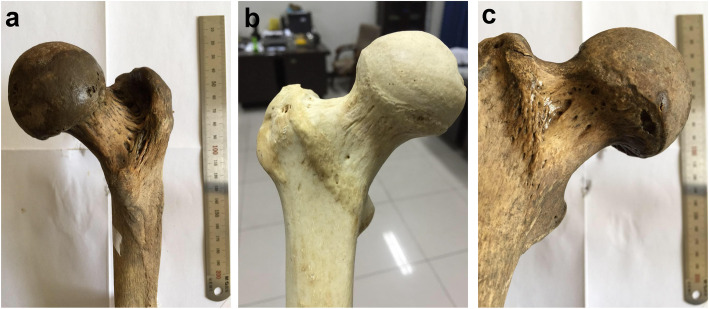
Osteo-line classification. **a** A-type Osteo-line. **b** B-type Osteo-line. **c** C-Type indicates no Osteo-line on femur neck

After the observation of the 89 specimens, we found that the Osteo-line was more obvious on the greater trochanter side. In the continuation from the greater trochanter side to the lesser trochanter side, there were 3 types of the Osteo-line between the trochanter and the femur neck, which were marked as types A, B, and C (Fig. 2). Among them, the A-type is clearer, the B-type is fuzzy on the lesser trochanter side, and a transitional area is formed on the lesser trochanter side during the downward continuation of the greater trochanter side. C-type indicates no Osteo-line on the femur neck.

**Fig. 3:**
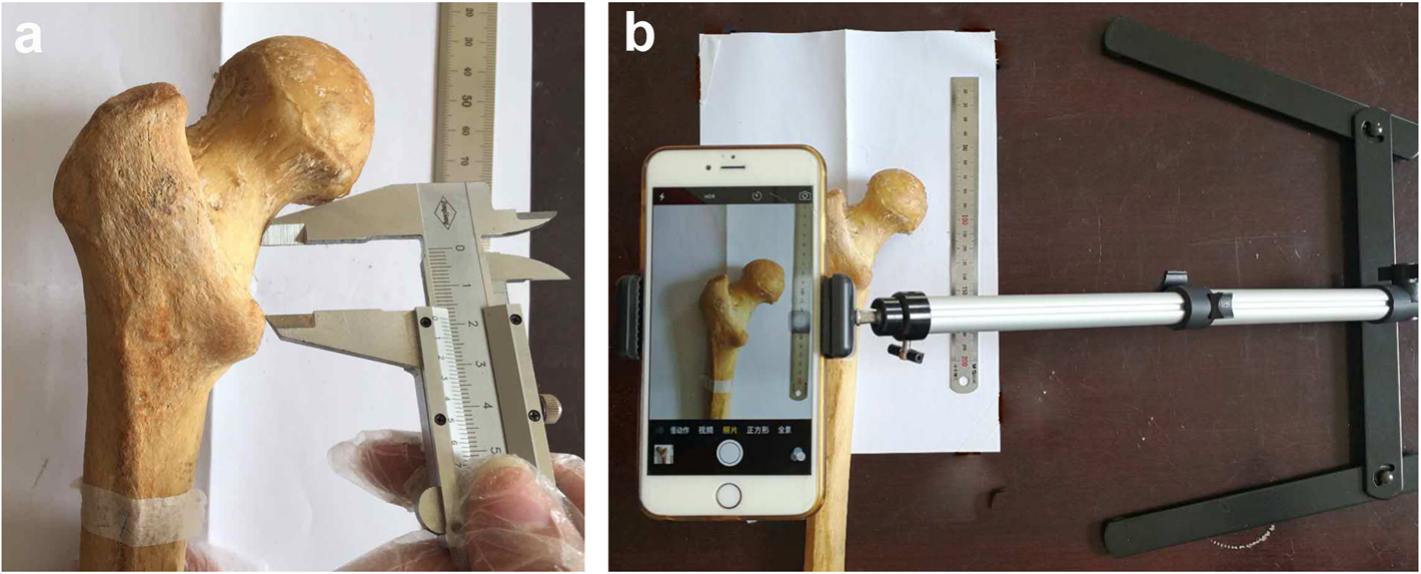
**a** Distance measurement between less tronchanter and the Osteo-line. **b** The facilities used to record the Osteo-line were showed

A vernier caliper was used to measure the distance “a” of the anterior Osteo-line on the femur neck between the lesser trochanter and the tip of the lesser trochanter and measure the distance “b” of the posterior Osteo-line on the femur neck between the lesser trochanter and the tip of the lesser trochanter (Fig. 3a). For the B-type Osteo-line, the extension line of Osteo-line on the greater trochanter side in the transition area was used as the reference line, and a vernier caliper was used to measure and record the upper and lower diameters of the attachment point of the iliopsoas muscle bundle at the lesser trochanter as “c”.

A mobile phone was installed on the multifunction digital camera remake frame. The femoral specimen was put in the test bench to make the long axis of the femoral neck parallel to the test bench, the steel ruler was put into the shooting field and set as the scale (Fig. 3b). The front and back views of the femur neck of the specimen were taken respectively, and the photo was numbered corresponding to the specimen number. The drawing tool (Photoshop) was used to measure and calculate the distance of the Osteo-line on the femur neck between the lesser trochanter and the tip of the lesser trochanter.

### Patients

From August 2015 to January 2019, a total of 278 patients who completed unilateral hip arthroplasty at the Third Hospital of Hebei Medical University were retrospectively included. Patients who accepted osteotomy via Osteo-line on the femur neck were defined as the experimental group (*n* = 139), and patients who accepted osteotomy via traditional method were defined as the control group (*n* = 139). The study was approved by the ethics committee of Third Hospital of Hebei Medical University (No. 2017-002-1), and signed informed consent was obtained from each patient.

Inclusion criteria were patients (1) who met the indications for hip arthroplasty surgery; (2) had primary hip arthroplasty surgery, had normal contralateral hip joint space, and had no obvious deformity of the lower limbs except the hip joint; (3) had non-pathological Gardenll I or IV type femoral neck fracture; (4) underwent ineffective conservative treatment; and (5) had clear consciousness and had no neurological or psychiatric diseases which affect their daily activities.

Exclusion criteria were patients who had (1) previous artificial hip and knee joint replacement surgery; (2) severe hip joint deformity; (3) hip joint infection; (4) bone tumor and other diseases; (5) congenital dysplasia of the hip; (6) dysplasia of the small trochanter; (7) revision surgery of the hip joint; (8) subcutaneous muscle tumor and infection; (9) preoperative surgery including serious underlying diseases, coagulation dysfunction, deep vein thrombosis, combined multiple organ infection and organ failure; and (10) neurological or mental illnesses that affect their daily activities.

### Surgical methods

Patients accepted osteotomy via Osteo-line on the femur neck for the experimental group. For the control group, the femoral distance 1.5 cm above the trochanter was retained for osteotomy by visual inspection.

### Comparison of the length of both lower limbs after operation

**Fig. 4:**
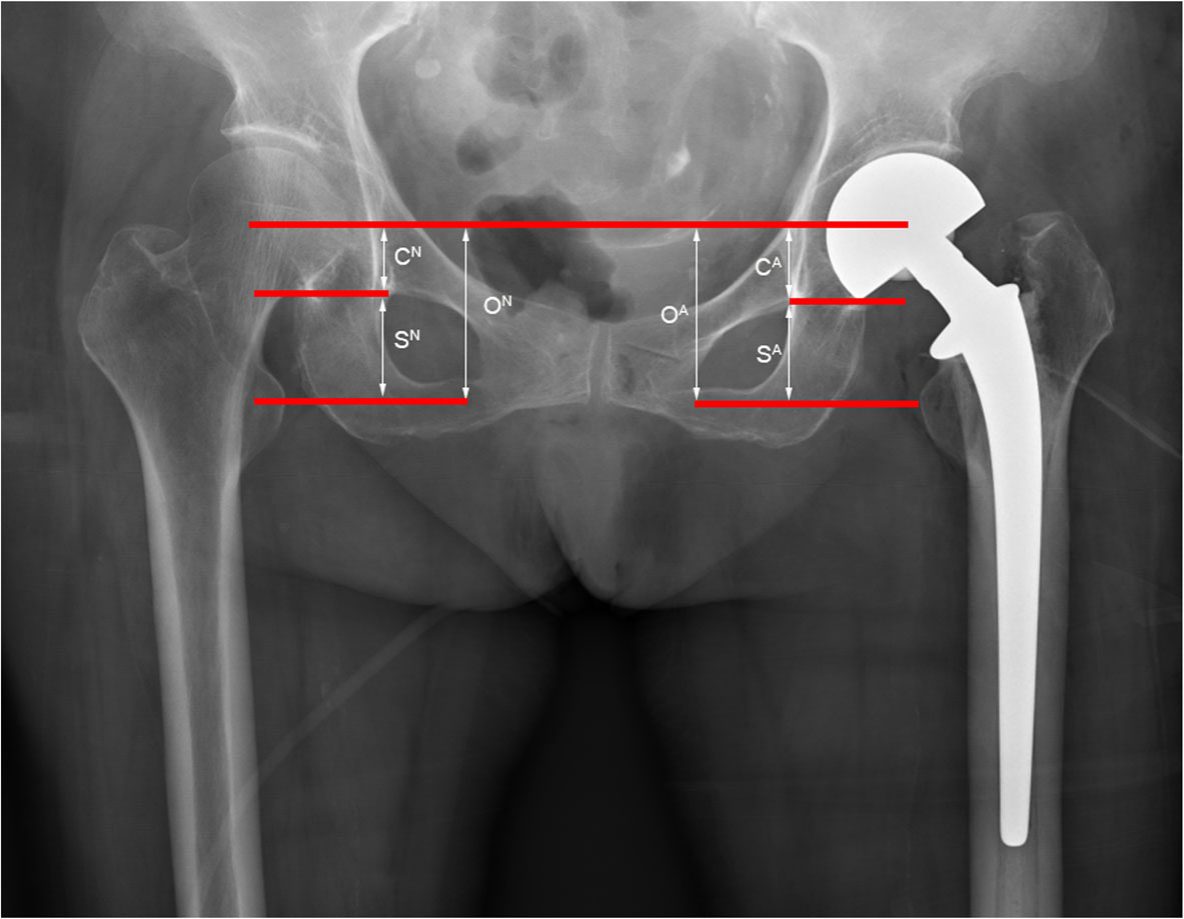
According to the postoperative X-ray, the drawing tool (Photoshop) was used to evaluate the unequal length of the lower limbs by the CFR-T-LT method. An initial reference line is drawn between the centers of femoral rotation, and two further lines are drawn parallel to it. The first at the level of the most inferior part of the acetabular teardrop to give measurement C, which corresponds to any inequality (C^A^-C^N^) due to the position of the cup. The second is at the level of the center of the lesser trochanter to give measurement S, which corresponds to inequality (S^A^-S^N^) due to position of the stem. The sum of the two is measurement O which corresponds to the overall leg length inequality (O^A^-O^N^)

According to the postoperative pelvic orthographic X-ray (scale bar = 120; pixels = 1 cm), the drawing tool (Photoshop) was used to evaluate the unequal length of the lower limbs by the CFR-T-LT method, as described by Anthony et al. [14] (Fig. 4). An initial reference line is drawn between the centers of femoral rotation, and two further lines are drawn parallel to it. The first is at the level of the most inferior part of the acetabular teardrop to give measurement C, which corresponds to any inequality (C^A^-C^N^) due to the position of the cup. The second is at the level of the center of the lesser trochanter to give measurement S, which corresponds to inequality (S^A^-S^N^) due to the position of the stem. The sum of the two is measurement O which corresponds to the overall LLD (O^A^-O^N^).

## Statistical analysis

Statistical analysis was performed using SPSS 20.0 software (IBM, USA). Values were presented as mean ± standard deviation (SD). LLD between the two groups were compared by independent samples *t* test. A *P* value of < 0.05 was considered statistically significant.

## Results

### Specimen observation results

Among the 89 specimens, the incidence of anterior Osteo-line on the femur neck was 75.28%, and the incidence of the posterior Osteo-line on the femur neck was 100%. Among the anterior Osteo-line on the femur neck, 14.61% were A-type Osteo-line, 60.67% were B-type Osteo-line, and 24.72% were C-type. Among the posterior Osteo-line on the femur neck, 38.20% were A-type Osteo-line, 61.80% were B-type Osteo-line, and there were no C-type.

The average distance “a” was 20.48 ± 1.79 mm. The average distance “b” was 20.42 ± 1.85 mm. The average distance “c” was 16.15 ± 2.13 mm. The distance from the upper edge of the iliopsoas muscle attachment point to the anterior Osteo-line on the femur neck during the operation (a-c/2) was 12.32 ± 1.51 mm. The distance from the upper edge of the iliopsoas muscle attachment point to the reflexed line of the posterior Osteo-line on the femur neck during the operation (b-c/2) was 12.34 ± 1.46 mm. The data measured by the photo was consistent with the result.

### Clinical application results

For the experimental group, of the 20 patients undergoing hemi-hip arthroplasty through the anterolateral approach, Osteo-line on the femur neck can be observed in 16 patients (80%). Of the 119 patients undergoing hemi-hip arthroplasty through the posterolateral approach, Osteo-line on the femur neck can be observed in 119 patients (100%). During the operation, it was found that the anterior and posterior Osteo-line on the femur neck were clearer than those on the femoral specimens.

Twenty-six cases had LLD greater than 1 cm (9.29%), including 2 cases in the experimental group and 24 cases in the control group. The average postoperative LLD in the experimental group (0.19 ± 0.38 mm) were significantly shorter than the control group (0.54 ± 0.51 mm) (*P* = 0.005) (Table 1). No cases had LLD greater than 2 cm.

**Table 1:** Comparison of postoperative LLD between the two groups

	Experimental group (*n* = 139)	Control group (*n* = 139)	*P* value
Postoperative LLD	0.19 ± 0.38 mm	0.54 ± 0.51 mm	0.005

*LLD* leg length discrepancy

## Discussion

LLD after THA remains a major problem [15]. Using the plane between 1.25 ± 0.25 cm above the tip of the lesser trochanter and the line to the base of the femoral neck on the greater trochanter side as the femoral neck osteotomy position in THA is subjective and leading to LLD [4]. We, in this study, found the incidence of Osteo-line on the femur neck is high, and patients who accepted osteotomy via Osteo-line on the femur neck can achieve shorter average postoperative LLD than the control group. In addition, using the Osteo-line as an anatomical landmark for femoral neck osteotomy can effectively simplify the surgical steps of THA and easy to learn and popularize, which can be used as a conventional femoral neck osteotomy in THA.

Our researchers found that during the transition of the cortical bone from the trochanteric part to the cortical bone of the femoral neck, an Osteo-line is naturally formed at the front and back of the base of the femoral neck. The top of Osteo-line (point A) is located exactly at the base of the femoral neck on the greater trochanter side, and the bottom of Osteo-line (point B) is located on the side of the lesser trochanter of the femur, and the Osteo-line is within about 1.25 ± 0.25 cm above the tip of the lesser trochanter. Using the Osteo-line as an anatomical landmark for femoral neck osteotomy can effectively simplify the surgical steps of hip arthroplasty. According to specimen measurement, the incidence of anterior Osteo-line on the femur neck was 80%, and the incidence of posterior Osteo-line on the femur neck was 100%. Moreover, the patients in the experimental group had LLD less than 7 mm, which is in line with the standard proposed by Austin et al. [16].

The advantages of performing osteotomy according to Osteo-line on the femur neck were as follows: (1) The osteotomy through Osteo-line is performed according to the anatomical structure of the femoral neck; therefore, the formulation of the surgical plan is more individualized. (2) No special auxiliary equipment, repeated measurement, and repeated osteotomy is needed, which make this method simple and shorten the operation time. (3) The osteotomy prosthesis is well matched with the proximal femur, which can avoid repeated correction of the osteotomy plane and re-injury. (4) It is not necessary to expose the lesser trochanter, and only the base of the femoral neck needs to be peeled off. After confirming the position of the Osteo-line, the operation can be completed without extending the surgical incision, which reduces intraoperative bleeding and surrounding soft tissue damage and ensures the stability of the joint. (5) This method is suitable for hip arthroplasty surgery that cannot measure the original femoral neck length. (6) This method simplifies the surgical technique and shortens the learning curve. However, there are some deficiencies of this method. The occurrence rate of the Osteo-line on the femur neck is not 100%. In addition, the existence of the B-type Osteo-line may also lead to wrong judgment by surgeons. This method is not available in patients without the Osteo-line or patients who lost the Osteo-line.

There were some limitations in this study. The number of femoral specimens observed in this study was small. Moreover, the retrospective design was another limitation. Therefore, large randomized RCTs remain to be studied in the future.

## Conclusion

The incidence of Osteo-line on the femur neck are high, and patients who accepted osteotomy via Osteo-line on the femur neck can achieve shorter average postoperative LLD than the control group. This method is easy to learn and popularize, which can be used as a conventional femoral neck osteotomy in hip arthroplasty.

## Abbreviations

LLD: Leg length discrepancy
THA: Total hip arthroplasty
